# Application of ^19^F NMR Spectroscopy for Content Determination of Fluorinated Pharmaceuticals

**DOI:** 10.1155/2017/9206297

**Published:** 2017-10-18

**Authors:** Alex O. Okaru, Tobias S. Brunner, Svenja M. Ackermann, Thomas Kuballa, Stephan G. Walch, Matthias Kohl-Himmelseher, Dirk W. Lachenmeier

**Affiliations:** ^1^Department of Pharmaceutical Chemistry, University of Nairobi, P.O. Box 19676-00202, Nairobi, Kenya; ^2^Chemisches und Veterinäruntersuchungsamt (CVUA) Karlsruhe, Weissenburger Strasse 3, 76187 Karlsruhe, Germany

## Abstract

A simple, rapid, and selective quantitative nuclear magnetic resonance spectroscopic method was evaluated for the determination of the content of fluorinated pharmaceuticals. ^19^F NMR spectra were either obtained in dimethylsulfoxide-*d*_*6*_ or aqueous buffer, using trifluoroacetic acid as internal standard. Quantification of 13 fluorine-containing pharmaceuticals spanning various pharmacological classes was accomplished using the proposed method. The method was found to be fit for purpose (interday precision 1.2% relative standard deviation) and may thus be applied for routine analysis and quality control of fluorine-containing pharmaceuticals due to its simplicity, nondestructive sample measurement, reliability, and high specificity. Therefore, ^19^F NMR may serve as a suitable analytical tool for the identification and selective determination of fluorinated pharmaceuticals used as reference materials and bulk samples.

## 1. Introduction

There is growing need for modern analytical techniques for analysis of fluorine-containing pharmaceutical substances including active pharmaceutical ingredients, brand and generic finished products, and also for the detection of potentially counterfeited pharmaceuticals. These new techniques are aimed not only at maximizing analytical throughput but also minimizing expenses while preserving acceptable method performance characteristics. Nuclear magnetic resonance spectroscopy (NMR) is such a technique whose scope of applications continues to expand. Indeed with the marked improvements on the sensitivity of NMR spectrometers, quantitative analytical applications have tremendously sprung up across many fields including foods, beverages [1–5], and pharmaceuticals [6]. NMR spectroscopy offers unparalleled rich information on samples, and, with rapid validated methods, high throughput can be achieved without destroying the sample [7]. Unlike the current chromatographic techniques that require reference standards, often expensive and unavailable in many laboratories, NMR spectroscopy enables quantification without necessity of a primary reference standard, thus lowering the cost of analysis [8, 9].

Traditionally, most of the NMR spectroscopic applications are based on ^13^C and ^1^H nuclides due to the relatively high abundance of carbon and hydrogen in natural compounds compared to phosphorus and fluorine. As an alternative nuclide, ^19^F offers the advantages of higher natural abundance compared to ^13^C and less risk of signal overlap compared to ^1^H since proton NMR shows a narrow range of the chemical shift (typically 0–10 ppm) and shows an increased spectral complexity due to coupling of neighbouring protons. Furthermore, ^19^F NMR has a broader chemical shift range (approx. 500 ppm) [10], which helps to avoid signal overlap and shows less interference from homonuclear coupling [11–13] and more importantly the solvent (mostly containing no fluorine) has no effect on the ^19^F NMR signal and as such solvent suppression is not necessary. This makes the technique uniquely suitable for quantification of fluorine-containing compounds in various matrices including finished pharmaceutical products. Currently over 200 medicines and one-third of the top-performing drugs on the market [14] contain fluorine atoms in their structure. This gives credence to the plausibility of using ^19^F NMR spectroscopy as an alternative analytical technique for assay of fluorinated drugs.

**Figure 1:**
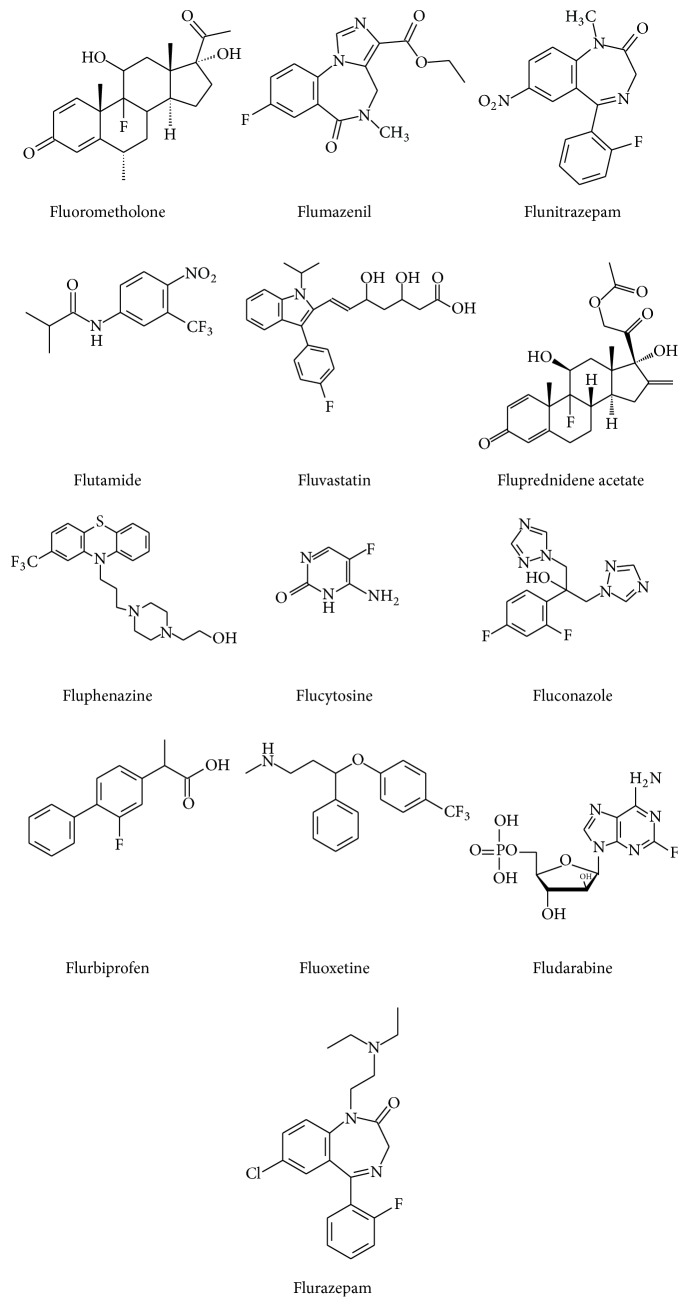
Chemical structures of the fluorine-containing pharmaceuticals studied.

Currently, various instrumental analytical methods based on chromatography, spectroscopy, and electrochemistry are used to determine some of these fluorine-containing compounds. Chromatographic techniques such as thin layer chromatography [15, 16], HPLC [17–20], gas chromatography or capillary electrophoresis [21], and spectrophotometric methods such as UV spectroscopy [17, 22] and electroanalytical techniques [23] have been utilized for purity determination of fluorinated compounds. These methods generally require the use of reference substances, expensive columns, and large volumes of organic solvents and in some cases laborious and time-consuming sample preparative steps. More often, the analysis takes a long time. NMR methods have been proven to be simple, reasonably rapid, and cost-effective in the long term compared to HPLC [24, 25]. Except for the determination of decomposition products of flurazepam dihydrochloride in various media at different temperatures by ^19^F NMR [26, 27], to the best of our knowledge, no single study has been published where ^19^F NMR spectroscopy has been applied for the content determination of fluorinated pharmaceuticals spanning across many pharmacological classes including anticancer drugs (fludarabine and flucytosine), antipsychotic agents (fluphenazine), anxiolytics (fluoxetine), sedative hypnotics (flumazenil and flunitrazepam), antiandrogens (flutamide), nonsteroidal anti-inflammatory agents (flurbiprofen), antifungal agents (fluconazole and flucytosine), and glucocorticoids (fluprednidene and fluorometholone) (Figure 1). The aim of this study is therefore to validate and apply a ^19^F NMR spectroscopic method for the determination of content of twelve fluorine-containing pharmaceuticals.

## 2. Experimental Section

### 2.1. Samples and Chemicals

The samples for analysis were fluorinated pharmaceuticals consisting of fluorometholone, flutamide, sodium flurbiprofen (USP-Standard, Rockville, MD, USA), flumazenil, flunitrazepam (Hoffmann-La Roche, Grenzach-Wyhlen, Germany), fluvastatin sodium hydrate (Hemofarm, Vrsac, Serbia), fluprednidene acetate (Alcon-Pharma, Freiburg, Germany), fluphenazine dihydrochloride (from the former pharmaceutical company Byk-Gulden, Konstanz, Germany), fluoxetine hydrochloride (Teva, Ulm, Germany), fludarabine phosphate (CRS-standard from EDQM, Strasbourg, France), flurazepam hydrochloride (Roche Diagnostics, Mannheim, Germany), flucytosine (Sigma-Aldrich, Taufkirchen, Germany), and fluconazole (Dr. Reddy's, Hyderabad, India). Proanalysis quality reagents, trifluoroacetic acid (TFA), and dimethylsulfoxide-*d*_*6*_ were obtained from Sigma-Aldrich (Taufkirchen, Germany).

### 2.2. Confirmation of Purity of Trifluoroacetic Acid (TFA) Using Ion Chromatography

The purity of TFA intended to be used as internal standard for ^19^F NMR spectroscopy was determined using a validated in-house ion chromatographic method. The separation of TFA was performed on a Metrohm system (Deutsche Metrohm GmbH & Co. KG, Filderstadt, Germany), containing an autosampler (Professional Sample Processor Mod. 858) connected to a stirrer Mod. 801 and Dosino Mod. 800 (10 mL) for degassing and stirring. The liquid TFA standard solution was transferred via a loop to the anionic column (Metrosep A Supp 5 150/4.0, Metrohm), maintained at 35°C in the chromatography Compact IC system (Mod. 881, Metrohm). The eluent consisted of 3.2 mM Na_2_CO_3_ and 2.4 mM NaHCO_3_ delivered at a flow rate of 0.7 mL·min^−1^. The injection volume was 20 *μ*L. All steps of ion chromatographic measurements were performed using Metrohm software MagIC NET Professional 3.1. The purity was found to be 99.83% by ion chromatography. For NMR measurements, 26.62 mg TFA was dissolved in 2 ml dimethylsulfoxide-*d*_*6*_ as internal standard solution.

### 2.3. NMR Analysis

Sample powder equivalent to 5 mg of fluorometholone, flumazenil, flunitrazepam, flutamide, fluvastatin sodium hydrate, and fluprednidene acetate were each dissolved in 1.0 ml dimethylsulfoxide-*d*_*6*_. Then to 500 *μ*l of the resultant solution, 100 *μ*l of TFA internal standard solution for referencing and quantitation (see Section 2.2) was added before NMR analysis. Similarly, sample powder equivalent to 5 mg of fluphenazine, fluoxetine, fludarabine, flurazepam, flucytosine, fluconazole, and flurbiprofen were prepared in 1.0 ml of buffer (pH 9.0) consisting of 1000 mg Li_2_CO_3_, 50 ml distilled water, 10.2 ml 1 M HCl, and 4 mg NaN_3_. For NMR measurements, 500 *μ*l of the resultant solution, 50 *μ*l of TFA, and 50 *μ*l TSP (sodium salt of 3-(trimethylsilyl)-propionate acid-*d*_*4*_)/D_2_O (for locking) were vortexed until the sample and internal standard were completely dissolved. The clear solution was transferred into an analytical NMR tube followed by acquisition of NMR spectra on a Bruker Avance III HD 400 NanoBay spectrometer (Bruker BioSpin, Rheinstetten, Germany) equipped with a 5 mm BBO probe using a Bruker Automatic Sample Changer (SampleXpress). The spectra were automatically acquired under the control of ICON-NMR (Bruker BioSpin, Rheinstetten, Germany). All ^19^F NMR spectra were recorded in the range of F −20.0 to −210.0 ppm (CF_3_COOH as reference, *δ* = −75.0 ppm) relative to TFA (*δ* = −0.0 ppm). The NMR probe was maintained at 300 K during the whole experiment. A delay time (*D*1) of 20 s was adopted to ensure full *T*1 relaxation, and pulse angle of 90° was used to provide maximum signal-to-noise ratio (S/N) and minimize the influence of off-resonance effect on the accuracy of ^19^F NMR measurement [28]. To maximize sensitivity, 512 scans were collected into 65536 data points over a spectral width of typically 95–125 ppm (for details see Tables 1 and 2). The Bruker pulse program, zgfhigqn, was used with a receiver gain (RG) of 203. All ^19^F NMR spectra were automatically phased and baseline corrected for accurate quantitative measurements using the Topspin 3.2 software package (Bruker BioSpin, Rheinstetten, Germany). Peak areas were obtained by electronic integration of the expanded regions around diagnostic resonances, using an integral limit of ±20 Hz around the corresponding signals. The percentage of error (reported in % relative standard deviation) was found to be less than 1%.

The purity of the analyte *P* was calculated using the following:

(1) $$\begin{array}{c} P=\frac{A_{SMP}}{A_{ISTD}}\cdot \frac{N_{ISTD}}{N_{SMP}}\cdot \frac{M_{SMP}}{M_{ISTD}}\cdot \frac{Q_{ISTD}}{Q_{SMP}}\cdot P_{ISTD}, \end{array}$$

where *A*_SMP_ and *A*_ISTD_ are the areas of selected signals for sample of fluorinated pharmaceutical and internal standard; *N*_SMP_ and *N*_ISTD_ are the number of fluorine atoms in the fluorinated pharmaceutical and internal standard; *M*_SMP_ and *M*_ISTD_ are the molar mass of sample of fluorinated pharmaceutical and internal standard; *Q*_SMP_ and *Q*_ISTD_ are amounts of fluorinated pharmaceutical and internal standard, respectively; *P*_ISTD_ is the purity of the internal standard as determined by ion chromatography.

To achieve acceptable accuracy and precision necessary for quantitative determination, the influence of the relaxation delay time, *D*1, on the S/N ratio of selected signals was investigated in the range of 5–35 s. Saturation of S/N was found in the range of 20–35 s. Therefore, for a short spectra acquisition run time, *D*1 = 20 s was preferred.

### 2.4. NMR Method Validation and Comparison

The proposed ^19^F NMR method was validated for selectivity, trueness, precision, and sensitivity according to the Eurachem criteria [29]. The trueness of the method was assessed in comparison with certifications of the manufacturers as well as with an independent method for some of the fluorinated compounds. HPLC analysis was conducted for fluorometholone, flumazenil, flunitrazepam, fluvastatin, and fluprednidene acetate (by using the specific methods for these substances from the admission dossier). The results were compared with the certificates of analysis from USP for flutamide or fluorometholone.

Selectivity of the proposed method was evaluated by determining the resolution between fluorine signals and other nuclei while precision was investigated by consecutive NMR measurements of a fluvastatin sample (including sample preparation) over 5 different days. The limit of detection (LOD) was manually estimated based on a small, but still integrable, signal in the region of the target resonance [30].

## 3. Results and Discussion

### 3.1. Method Optimization

Dimethylsulfoxide-*d*_*6*_ was selected as a solvent for the samples on account of its good dissolving properties while TFA was used as the internal standard. It is important to ascertain the purity of the reference substances before use in analysis since high purity is a prerequisite for a substance to be considered suitable for use as a standard in NMR spectroscopy. The purity of TFA used as an internal standard was established by ion chromatography to be 99.83%, which is considered adequate for use in our analytical purpose. TFA was selected as the internal standard for the purity assessment of the fluorine-containing pharmaceuticals on account of its good solubility in dimethylsulfoxide-*d*_*6*_ and also its sharp distinct signal at *δ*  −75.0 ppm (3F, s), which is well separated from the compounds being tested. Normally, the number of neighbouring protons influences the multiplicity of the ^19^F signal observed. However, to avoid this effect, proton decoupling was applied, so that each ^19^F signal appears as a singlet, which simplifies the spectra.

Experimental parameters for ^19^F NMR analysis of fluorinated pharmaceuticals were selected to optimize the NMR method with respect to accuracy, precision, and analysis time. The optimized measurement parameters were established as TD = 65536; number of scans (NS) = 512, *D*1 = 20 s; RG = 203. Longitudinal relaxation delay of the fluorine compounds was determined by the inversion recovery pulse sequence method, using *T*1/*T*2 Relaxation Bruker program which fitted the data to the exponential equation:

(2) $$\begin{array}{c} I=I_{0}+P\exp\left(\;-\tau /T1\;\right), \end{array}$$

where* I *is the intensity of the compound of interest resonance at the inversion delay time*τ*and *I*_0_ at the equilibrium state and* P* is a constant. The ^19^F NMR signals selected for quantitative purposes and the selected spectral widths (SW) are shown in Tables 1 and 2. ^19^F NMR spectra showed distinct, well separated, nonoverlapping signals that were selected for quantitation.

### 3.2. NMR Validation and Applicability

The selectivity of the NMR method was demonstrated since there was no signal overlap with other components. Acceptable precision was established for a fluvastatin sample with intraday and interday relative standard deviations (coefficient of variation) of 0.9 and 1.2%, respectively. The limit of detection was less than 0.1 g/100 g in 27 evaluated spectra (average limit of detection 0.06 g/100 g). Trueness was similarly demonstrated since the assay values as determined using NMR spectroscopy and HPLC were <5%.

The purity of 13 fluorine-containing pharmaceuticals was measured using the proposed ^19^F NMR method (Tables 3 and 4). Supplementary Figures S1–S13 show the ^19^F NMR spectra of all investigated compounds (see Supplementary Material available online at https://doi.org/10.1155/2017/9206297). A potential shortcoming of ^19^F NMR is that, due to the wide chemical shift dispersion, a single excitation pulse cannot produce a uniform excitation over the entire range of the chemical shift region of interest [28]. To overcome this problem usually two measurements have to be performed: one overview experiment over the whole range to identify the optimal sweep width and then a second experiment for quantification in this range. Additionally, the internal standard should have a similar chemical shift as the signal of interest.

The reliability of the results obtained by the ^19^F NMR spectroscopic method was tested by comparing them to the data obtained using HPLC method and certifications of manufacturers. The results are presented in Tables 3 and 4. In all samples, small differences were noted in the content of the fluorinated pharmaceuticals determined through the NMR method compared with HPLC method and as such the two methods can be equally applied in the analysis of fluorinated pharmaceuticals. Only one inconsistency was observed during our experiments: the spectrum of flurazepam (Figure S13) contains two distinct signals, which could be due to hydrolytic decomposition in acidic water solution previously observed in ^19^F NMR leading to a 44 : 56 ratio between flurazepam and its ring-opened form after 24 h equilibrium [25]. In the current study, the combined integral of both signals was used for quantification, but further analysis such as using LC/MS/MS is required for clarification.

Although proton NMR has been used for quantitative determination of pharmaceutical compounds in different matrices, there are few official applications [25]. The USP, for example, prescribes the use of NMR for assay of amylnitrite isomers. The European Pharmacopoeia uses NMR for the characterization of impurities in hydroxypropylcyclodextrin and poloxamer. Generally, the pharmacopoeia uses NMR spectroscopy mainly for the identification of drugs such as low molecular weight (LMW) heparins, tobramycin, and hydrocortisone sodium phosphate [25]. Additionally, reagents such as adenosine and butoxycaine are identified using both ^1^H NMR and ^13^C NMR spectroscopy. However, no compendium prescribes the use of ^19^F NMR for any assay. Therefore, our research expands the applicability of ^19^F NMR spectroscopy beyond purity determination to routine assays with appreciable sensitivity. However, to be used in practice, it would be necessary to extend the domain of application of the method for fluorine-containing drugs to expand the database. To the best of our knowledge, this study is the largest evaluation of fluorinated pharmaceuticals samples by ^19^F NMR spectroscopy.

## 4. Conclusion

The proposed ^19^F NMR spectroscopic method demonstrated adequate accuracy, simplicity, and reproducibility for a rapid routine analysis and quality control of important compounds of fluorine-containing pharmaceuticals. The ^19^F NMR method was successfully applied to determine the purity of fluorinated pharmaceuticals. The direct relationship between the peak area and number of fluorine nuclides was exploited. Due to the lack of interferences or signal overlap, ^19^F NMR offers adequate specificity for quantification of pharmaceuticals without preceding extensive sample preparation. Additionally, ^19^F NMR spectroscopy combines the speed, simplicity, and low cost per analysis. No reference standard of the analyte is required for quantification and also the method is nondestructive.

## Supplementary Material

Supplementary Figures S1-S13 (19F NMR spectra of all investigated compounds).

## Figures and Tables

**Table 1:** Spectral widths of DMSO-*d*_*6*_ soluble compounds.

Compound	Signal, ppm	Spectral width SW, ppm	Transmitter frequency offset O1, ppm
Fluorometholone	−166	171	121
Flumazenil	−114	119	95
Flunitrazepam (Rohypnol)	−114	119	95
Flutamide	−58	95	68
Fluvastatin sodium hydrate	−115	120	95
Fluprednidene acetate	−164	170	120

**Table 2:** Spectra widths and buffer pH of buffer soluble compounds.

Compound	Buffer pH	Signal, ppm	Spectral width SW, ppm	Transmitter frequency offset O1, ppm
Fluphenazine dihydrochloride	1.90	−62	95	−68
Fluoxetine HCl	1.90	−61	95	−68
Fludarabine phosphate	1.90	−52	105	−63
Flurazepam monohydrochloride	1.90	−111	121	−95
		−113		
5-Fluorocytosine	1.90	−169	175	−123
Fluconazole	1.90	−108	117	−93
		−110		
Sodium flurbiprofen dihydrate	9.0	−119	125	−98

**Table 3:** Analytical results for DMSO-*d*_*6*_ soluble fluorinated pharmaceuticals.

Compound	Purity (% w/w)		
	^19^F NMR	HPLC	USP-certificate
Fluorometholone	95.9	100.0	100.0
Flumazenil	94.9	99.8	n.p.
Flunitrazepam (Rohypnol)	98.8	99.8	n.p.
Flutamide	98.2	n.d.	98.0–101.0
Fluvastatin sodium hydrate	83.0	84.6	n.p.
Fluprednidene acetate	98.1	99.7	n.p.

n.p.: not provided, n.d.: not determined.

**Table 4:** Analytical results for buffer-soluble compounds.

Compound	Purity (% w/w)
	^19^F NMR^a^
Fluphenazine dihydrochloride	88.8
Fluoxetine HCl	84.0
Fludarabine phosphate	89.1
Flurazepam monohydrochloride	86.5
5-Fluorocytosine	82.8
Fluconazole	86.2
	85.9
Sodium flurbiprofen dihydrate	89.3

^a^Comparison HPLC data or USP certificates not available for these compounds.
