# Supplementary material for: Application of 19F NMR Spectroscopy for Content Determination of Fluorinated Pharmaceuticals
Source: J Anal Methods Chem. 2017 Oct 18;2017:9206297. doi: 10.1155/2017/9206297 (PMC5664369; doi:10.1155/2017/9206297)
Supplement: Supplementary file 1 — Supplementary Figures S1-S13 (19F NMR spectra of all investigated compounds). [file 9206297.f1.pdf]

SUPPLEMENTARY MATERIAL

Application of 19F NMR spectroscopy for content determination of fluorinated pharmaceuticals

Alex O. Okaru[1], Tobias S. Brunner[2], Svenja M. Ackermann[2], Thomas Kuballa[2], Stephan G. Walch[2], Dirk W. Lachenmeier[2]*

[1]*Department of Pharmaceutical Chemistry, University of Nairobi, P.O. Box 19676-00202 Nairobi, Kenya.*

[2]*Chemisches und Veterinäruntersuchungsamt (CVUA) Karlsruhe, WeissenburgerStrasse 3, 76187 Karlsruhe, Germany*

**E-mail:* lachenmeier@web.de.

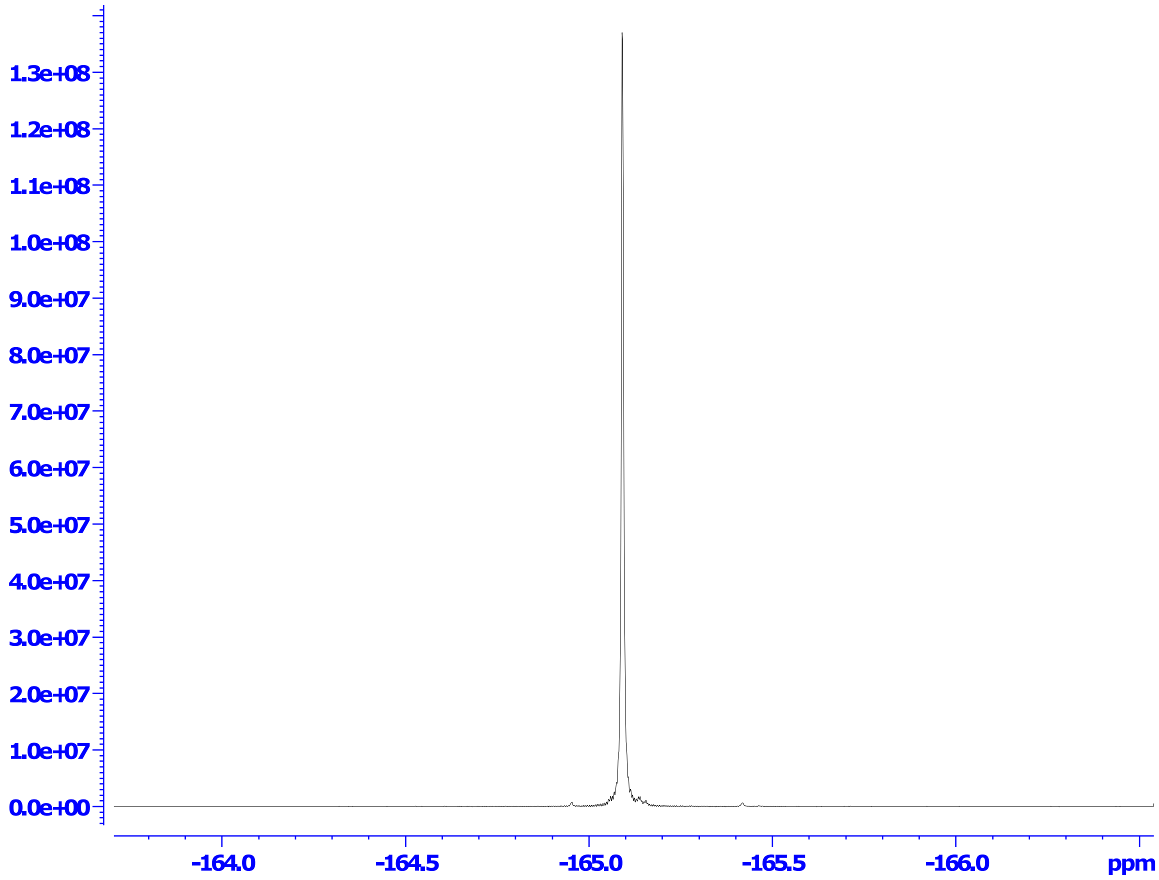

FIGURE S1: ^{19}F NMR spectrum of fluorometholone

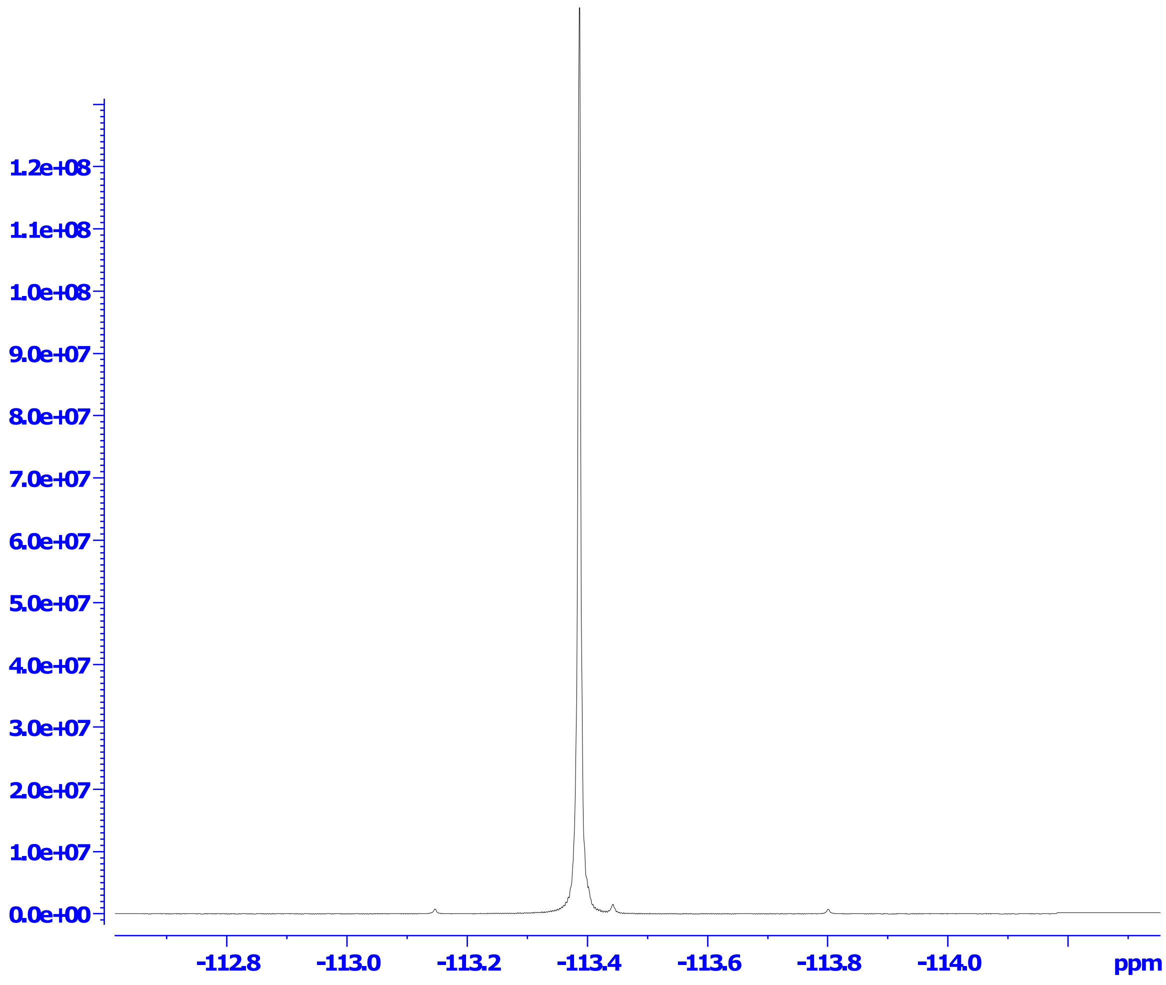

FIGURE S2: ^{19}F NMR spectrum of flumazenil

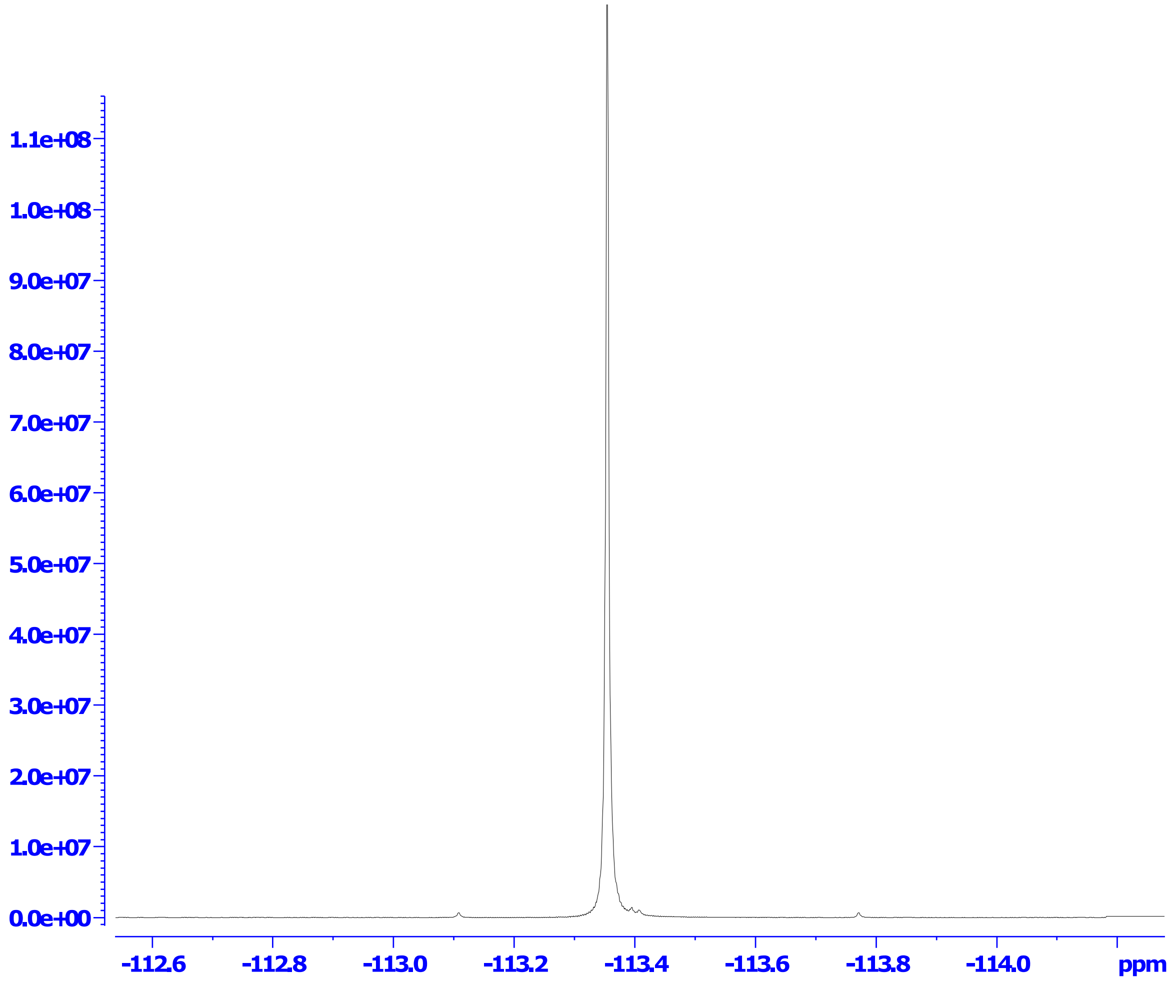

FIGURE S3: ^{19}F NMR spectrum of flunitrazepam

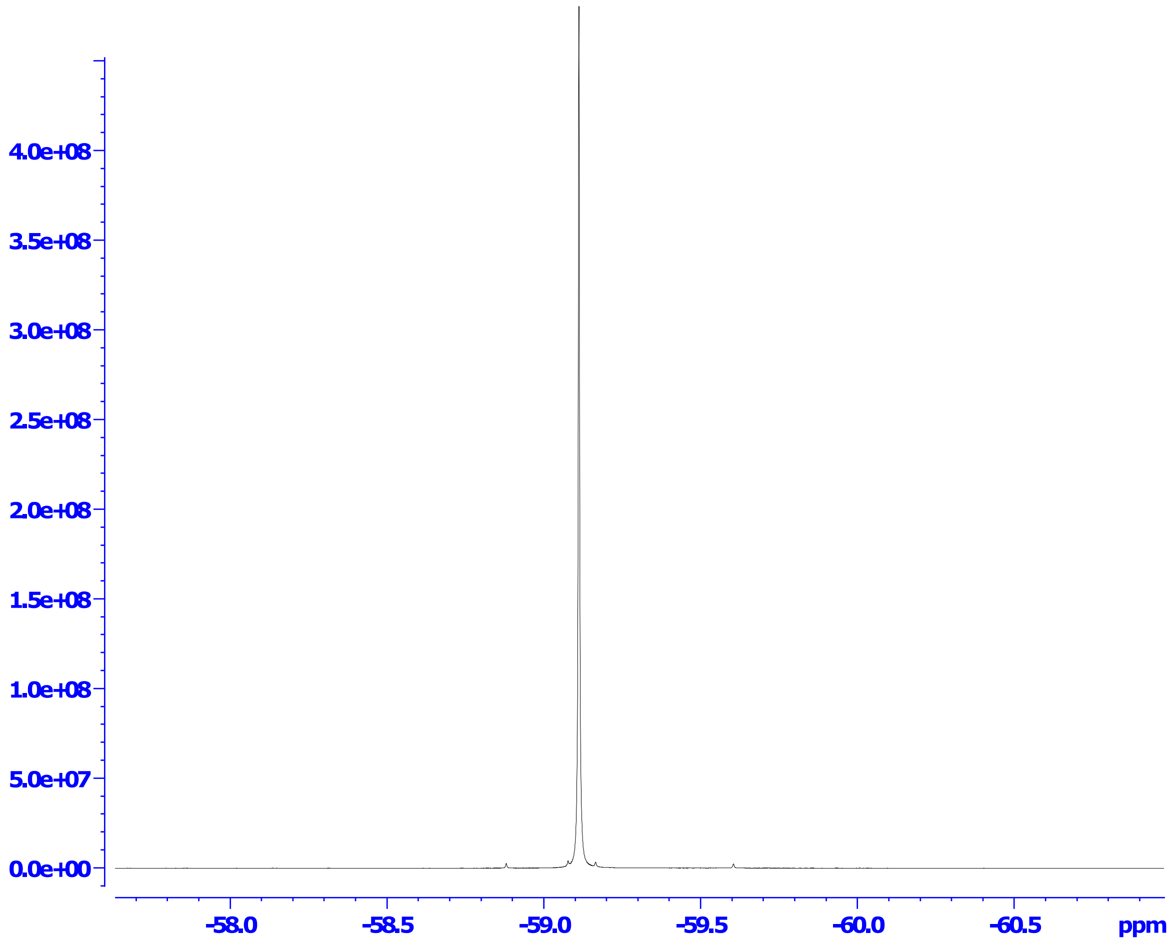

FIGURE S4: ^{19}F NMR spectrum of flutamide

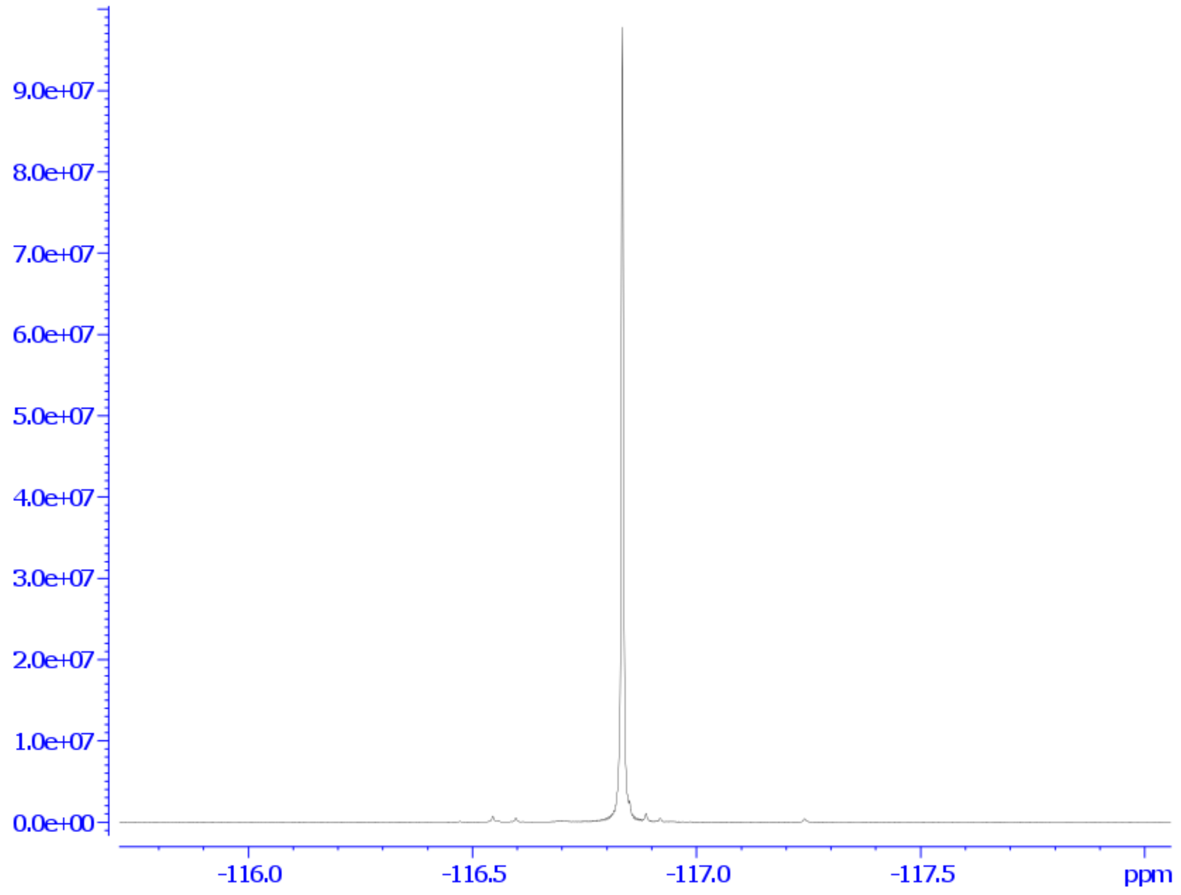

FIGURE S5: ^{19}F NMR spectrum of fluvastatin

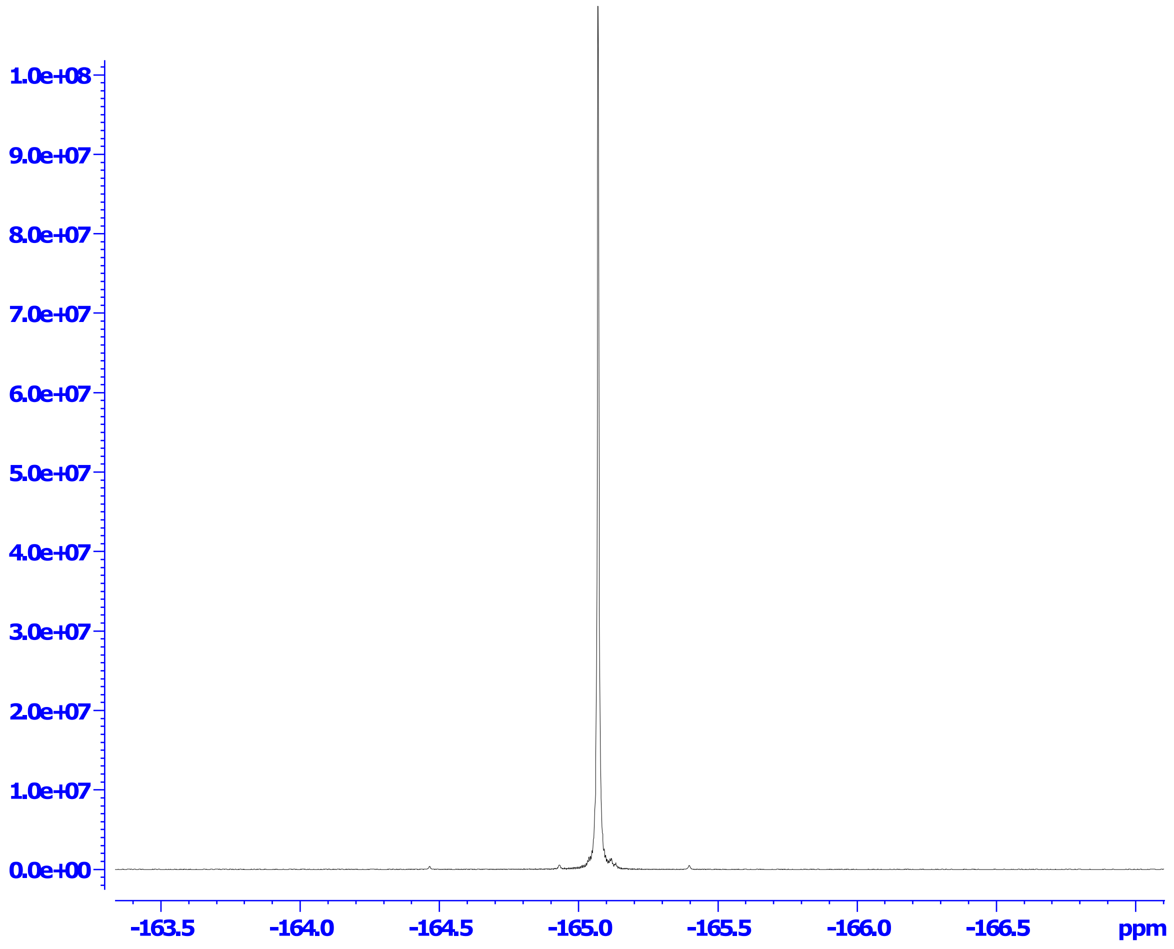

FIGURE S6: ^{19}F NMR spectrum of fluprednidene acetate

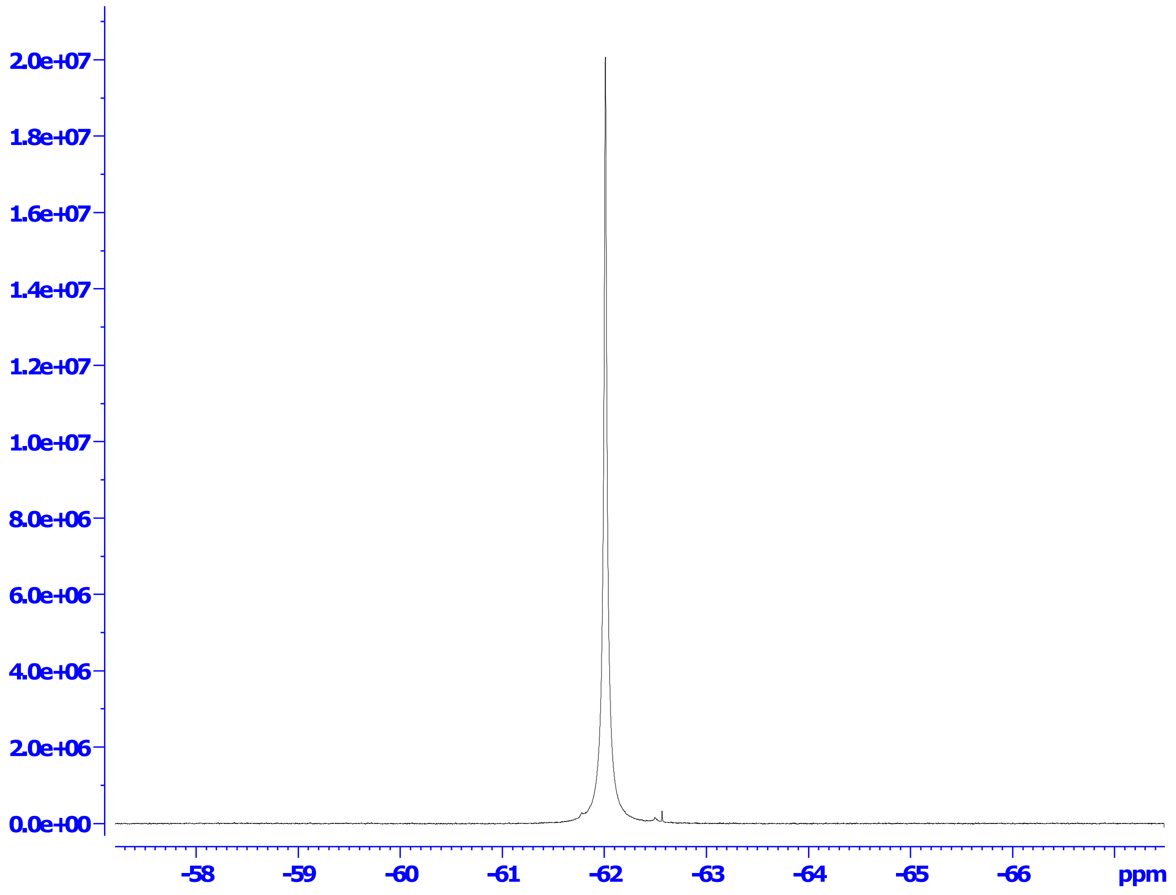

FIGURE S7: ^{19}F NMR spectrum of fluphenazine

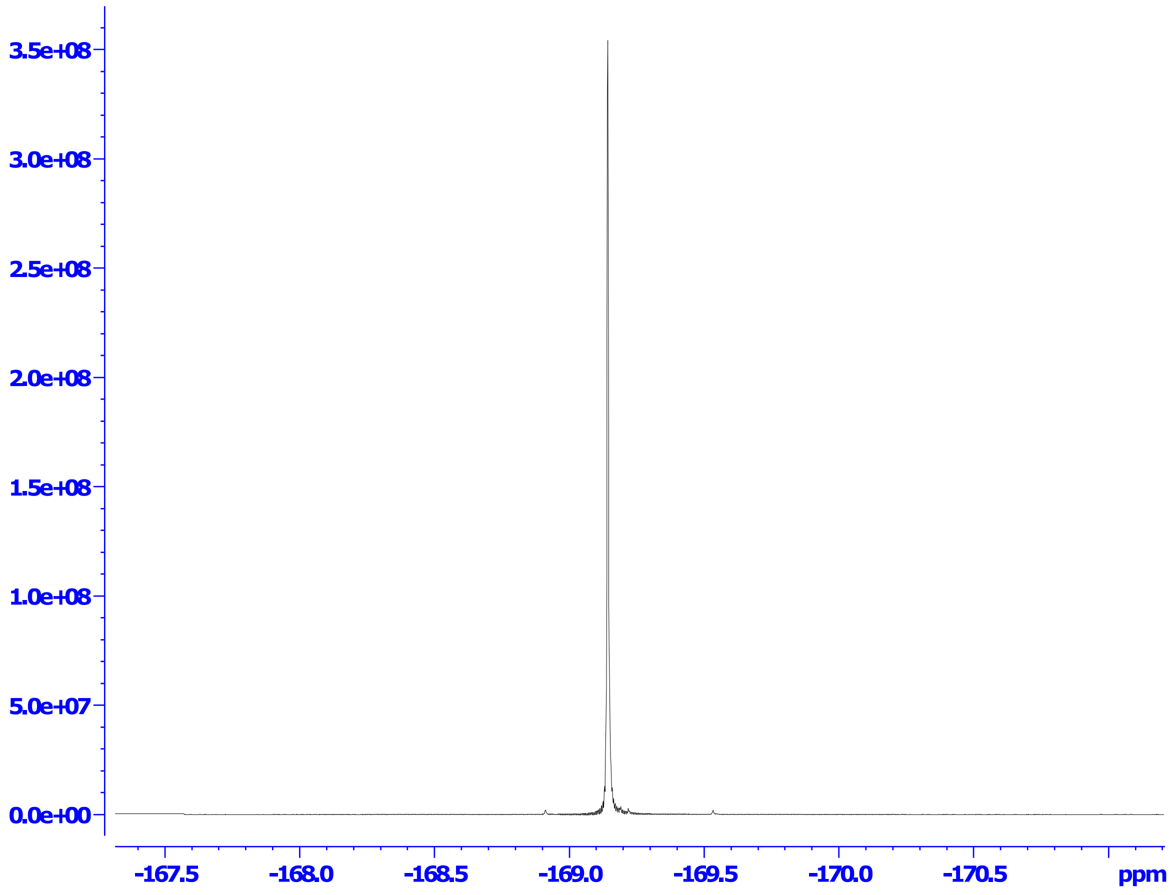

FIGURE S8: ^{19}F NMR spectrum of flucytosine

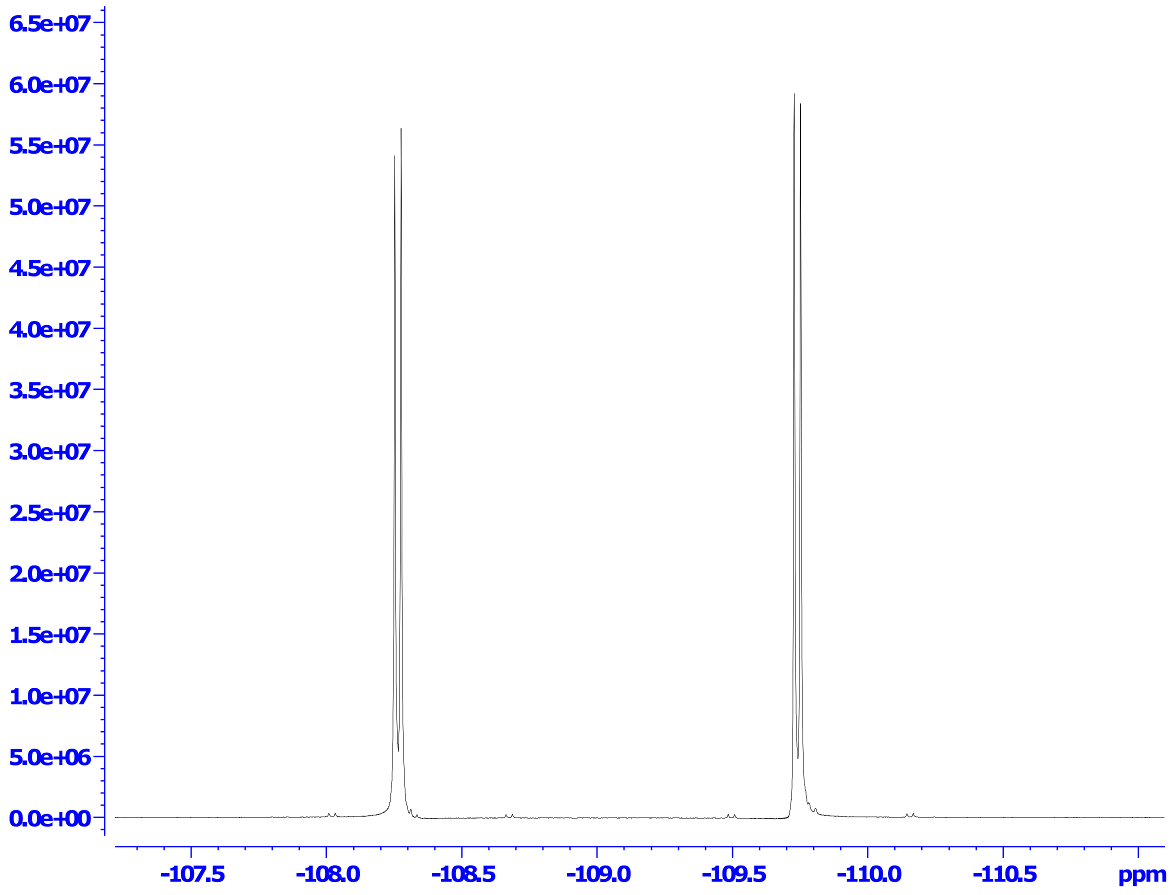

FIGURE S9: ^{19}F NMR spectrum of fluconazole

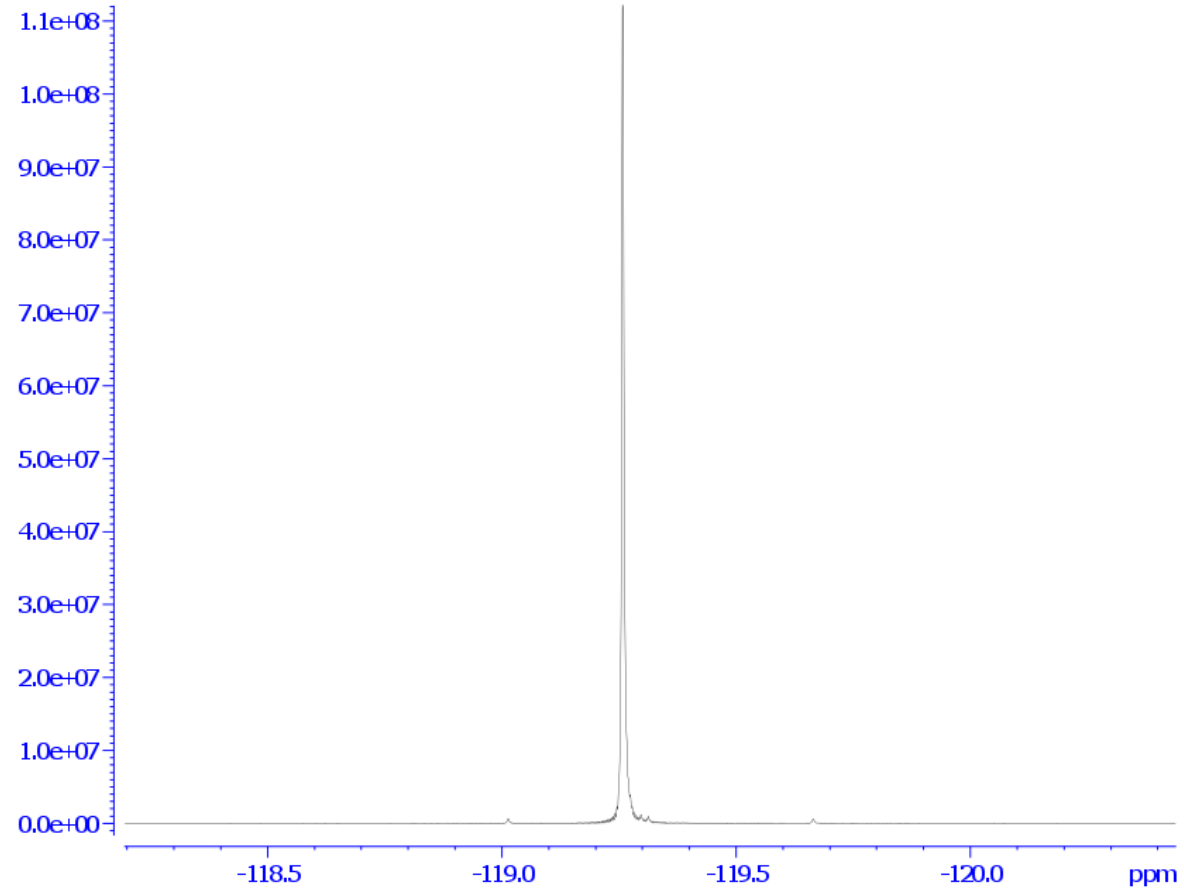

FIGURE S10: ^{19}F NMR spectrum of flurbiprofen

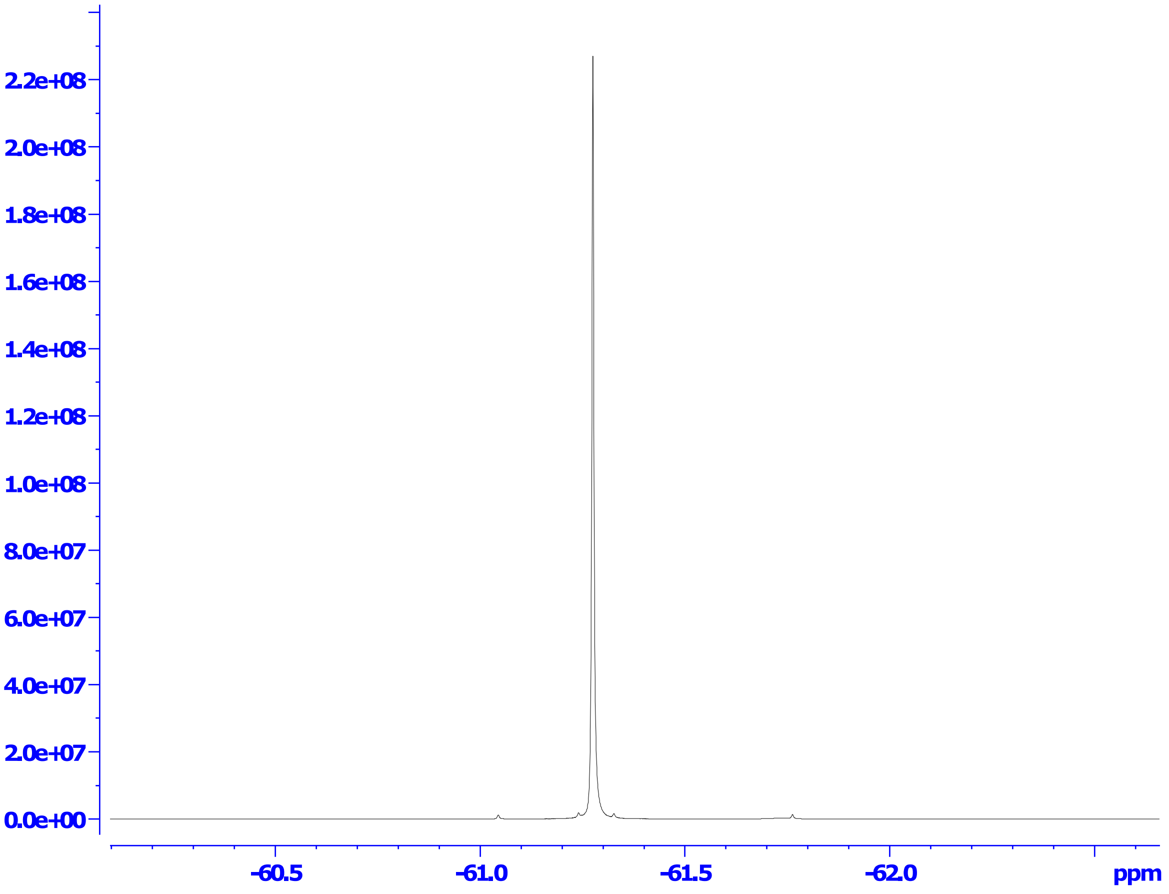

FIGURE S11: ^{19}F NMR spectrum of fluoxetine

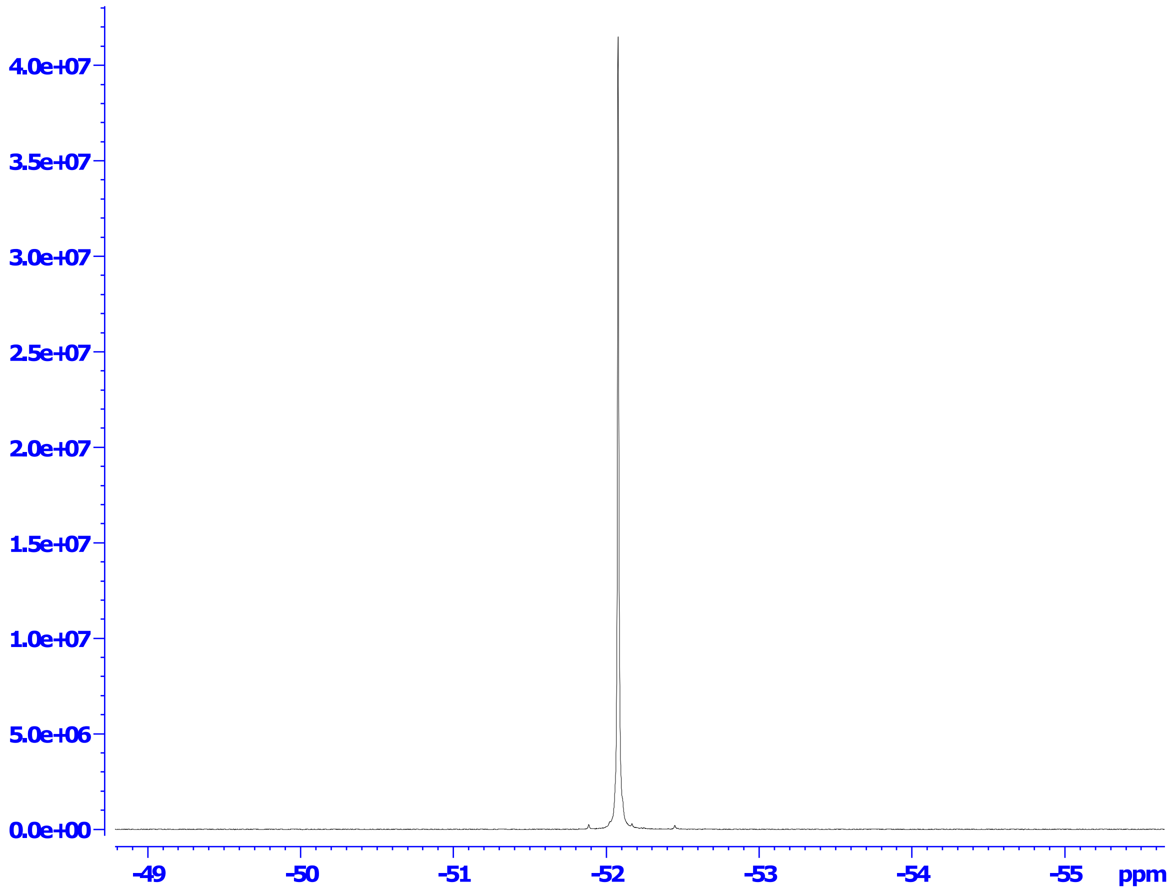

FIGURE S12: ^{19}F NMR spectrum of fludarabine

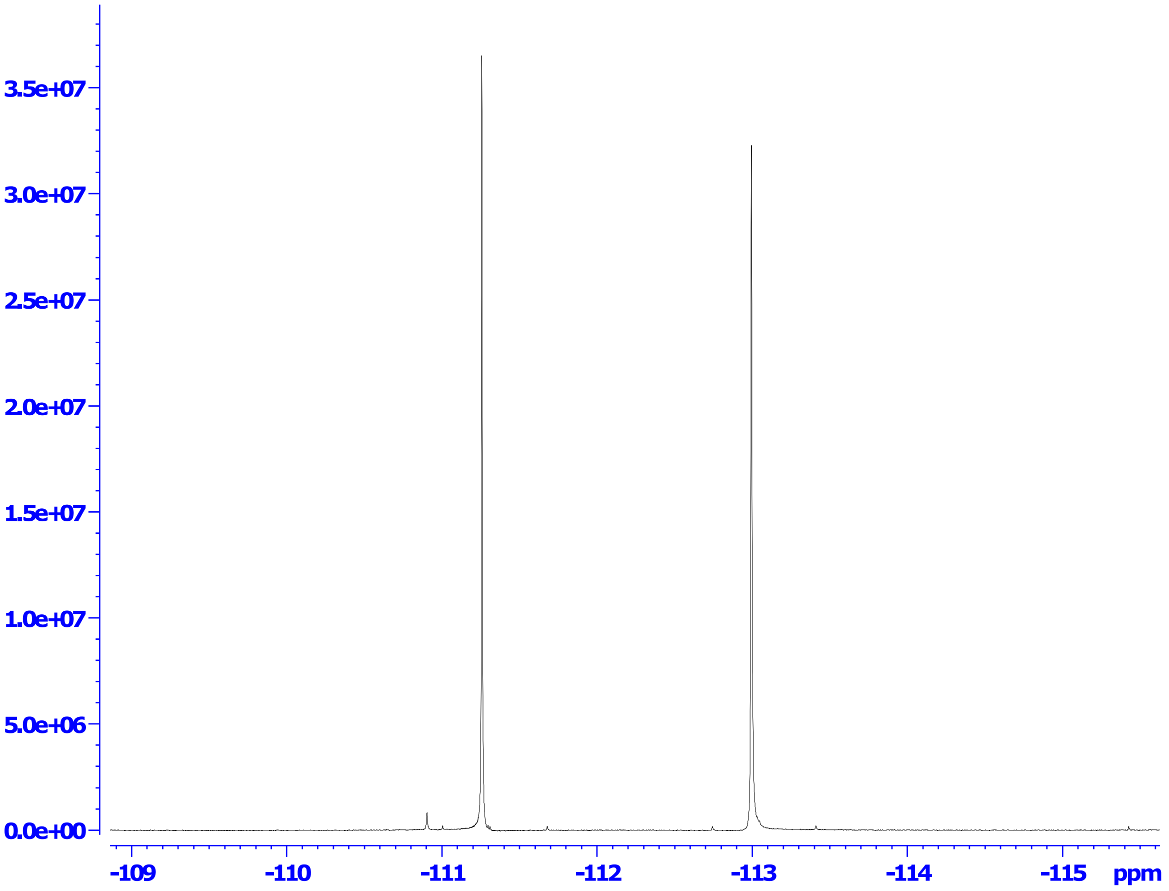

FIGURE S13: ^{19}F NMR spectrum of flurazepam
